# Brown Adipose Tissue: A New Potential Target for Glucagon-like Peptide 1 Receptor Agonists in the Treatment of Obesity

**DOI:** 10.3390/ijms24108592

**Published:** 2023-05-11

**Authors:** Tim Hropot, Rok Herman, Andrej Janez, Luka Lezaic, Mojca Jensterle

**Affiliations:** 1 Department of Pediatric Endocrinology, Diabetes and Metabolic Diseases, University Children’s Hospital, 1000 Ljubljana, Slovenia; tim.hropot@gmail.com; 2 Department for Endocrinology, Diabetes and Metabolic Diseases, University Medical Centre Ljubljana, 1000 Ljubljana, Slovenia; rokherman2@gmail.com (R.H.); andrej.janez@kclj.si (A.J.); 3 Department of Internal Medicine, Faculty of Medicine, University of Ljubljana, 1000 Ljubljana, Slovenia; luka.lezaic@kclj.si; 4 Department for Nuclear Medicine, University Medical Centre Ljubljana, 1000 Ljubljana, Slovenia

**Keywords:** obesity, weight loss, brown adipose tissue, glucagon-like peptide-1 receptor agonists, energy expenditure

## Abstract

Adipose tissue can be divided into white adipose tissue (WAT), brown adipose tissue (BAT), and beige adipose tissue, according to the differences in morphology. WAT acts as a buffer for increased energy intake and decreased energy expenditure during the development of obesity, resulting in visceral and ectopic WAT accumulation. These WAT depots are strongly associated with chronic systemic inflammation, insulin resistance, and cardiometabolic risk related to obesity. They represent a primary weight loss target in anti-obesity management. Second-generation anti-obesity medications glucagon-like peptide-1 receptor agonists (GLP-1RAs) cause weight loss and improve body composition by reducing visceral and ectopic fat depots of WAT, resulting in improved cardiometabolic health. Recently, the understanding of the physiological significance of BAT beyond its primary function in generating heat through non-shivering thermogenesis has been expanded. This has raised scientific and pharmaceutical interest in the manipulation of BAT to further enhance weight reduction and body weight maintenance. This narrative review focuses on the potential impact of GLP-1 receptor agonism on BAT, particularly in human clinical studies. It provides an overview of the role of BAT in weight management and highlights the need for further research to elucidate the mechanisms by which GLP-1RAs affect energy metabolism and weight loss. Despite encouraging preclinical data, limited clinical evidence supports the notion that GLP-1RAs contribute to BAT activation.

## 1. Introduction

The prevalence of obesity and its associated health complications is increasing worldwide, presenting a significant global health challenge. The World Health Organization (WHO) estimated that the worldwide prevalence of obesity nearly tripled between 1975 and 2016. Overall, about 13% of the world’s adult population was obese in 2016 [1]. The WHO regional obesity report for Europe in 2022 states that almost 60% of adults are overweight or obese. Furthermore, the report reveals that nearly one in three school-aged children are overweight or obese [2]. The alarming numbers of obesity prevalence are concerning, given that obesity is associated with increased mortality [3,4]. Over the last decades, it has become clear that obesity encompasses more than just an increase in body mass with a plethora of distinct comorbidities now combined in term adiposity-based chronic diseases that significantly impair health and also confer profound economic burden, mental health problems, unemployment, and decreased quality of life [5,6,7,8,9,10].

The fundamental pathophysiological driver behind obesity is chronic energy imbalance driven by a dysregulation in interactions involving satiety factors and the central nervous system, which leads to increased caloric intake and excess adipose tissue mass [10]. The characterization of obesity as a chronic, complex, adiposity-based disease is also emphasized in the American Association of Clinical Endocrinologists and American College of Endocrinology (AACE/ACE) comprehensive clinical practice guidelines for medical care of patients with obesity [11]. With the increased focus on adipose tissue, the efficacy of various weight loss interventions on body composition and adipose tissue distribution has gained an essential role in the determination of weight loss success [12]. Fat accumulation in ectopic tissues and the visceral adipose depots has been linked to the unfavorable metabolic profile, inflammation, and insulin resistance and is now positioned as a primary target of adipose tissue reduction [13,14]. However, over recent years, few research groups started to hypothesize about the critical role of brown adipose tissue (BAT) in glucose metabolism and total body energy expenditure [15]. BAT activation is debated as a potential therapeutic target for weight loss [16], and recent studies have shown that BAT activity is seemingly intact in obese individuals [17].

The advent of novel, second-generation anti-obesity medications, from currently approved glucagon-like peptide 1 receptor agonists (GLP-1RAs) to new agents in the pipeline, now provide powerful tools for the management of adiposity-based chronic diseases. Nevertheless, the entire field is keenly anticipating further clinical data on their efficacy and safety [10,18,19]. The mechanisms of action of those agents in weight loss are multi-layered, involving the central nervous system and peripheral tissues. Their potential to activate BAT has become of particular interest following encouraging initial results [20,21].

This narrative review aims to explore how GLP-1RAs potentially affect BAT, primarily focusing on human clinical studies while also reviewing preclinical data and providing a brief overview of the role of BAT in weight management. We searched MEDLINE using the search terms “GLP-1 receptor agonist“, “exenatide”, “lixisenatide”, “liraglutide”, “dulaglutide”, “albiglutide”, and “semaglutide” in conjunction with “brown adipose tissue“. Our inclusion criteria included clinical trials on human subjects, studies published in English, and studies assessing BAT changes or activation associated with GLP-RA treatment. No strict exclusion criteria were implemented regarding the number of study participants or study duration. The last search was conducted on 6 March 2023. Our search results revealed two studies meeting our inclusion and exclusion criteria, which are discussed in detail below. Our findings could provide insight into understanding the role of BAT when using GLP-1RAs to treat obesity. Lastly, we discuss the possibilities and limitations of measuring BAT as an outcome in future study designs for weight loss interventions.

## 2. Adipose Tissue Subtypes

Adipose tissue is viewed as a passive tissue that mainly served to store excess energy in the form of fat. However, it has become increasingly apparent that it also plays a significant role in metabolic homeostasis through endocrine signaling [22]. Adipose tissue can be divided into white adipose tissue (WAT), BAT, and beige adipose tissue, according to the morphological differences [23].

WAT accounts for approximately 10–20% of total body weight in lean adults, making it one of the body’s largest organs [24]. White adipocytes contain a single, large lipid droplet and have few mitochondria, primarily functioning as energy storage and endocrine tissue [25]. WAT can be loosely divided by location into subcutaneous and visceral WAT. In healthy, lean individuals, subcutaneous WAT represents approximately 80% of all adipose tissue. Given its physiological capability of excess lipid storage, subcutaneous WAT acts as a physiological buffer during a surplus in energy intake and a decrease in energy expenditure [23]. In addition, subcutaneous WAT, located beneath the skin, serves multiple other functions, such as insulation to prevent heat loss, protection against external mechanical stress, and defending against dermal infections [26]. During obesity development, WAT mass increases ectopically in areas including the omentum, mesenterium, and retroperitoneum, together encompassing visceral WAT. Visceral WAT is highly metabolically active, continuously releasing free fatty acids into the portal circulation, thus being associated with features of the metabolic syndrome [23].

Historically, BAT was believed to be present only in small and hibernating mammals and, to a certain degree, in human infants [23]. However, due to advances in medical imaging techniques, namely, fluorine-18 fluorodeoxyglucose positron emission tomography coupled with CT (^18^F-FDG-PET/CT), BAT was also observed in adults [16,27]. BAT depots are present in at least six anatomic regions in human adults, which include the cervical, axillary, supraclavicular, mediastinal, paraspinal, and abdominal regions [28], with BAT representing only approximately 1–2% of the entire adipose mass [29]. 

The primary physiological function of BAT is to maintain body temperature during cold exposure by generating heat through non-shivering thermogenesis [16]. In infants, the activation of non-shivering thermogenesis is crucial for the maintenance of normal body temperature, given their unfavorable surface-to-volume ratio [30]. Non-shivering thermogenesis in BAT is possible due to the specific characteristics of brown adipocytes. In contrast to white adipocytes, brown adipocytes contain numerous small lipid droplets, have a high mitochondrial density, and, most importantly, express high levels of uncoupling protein 1 (UCP1). The high iron content of mitochondria and their abundance in brown adipocytes give BAT its characteristic color [25]. Upon cold exposure, afferent signals from skin thermoreceptors are transmitted to the brain, exciting the sympathetic nervous system and thus stimulating sympathetic outflow to BAT, resulting in noradrenaline release from neural varicosities and interacting with adrenergic receptors on brown adipocytes. This interaction induces intracellular lipolysis and, in turn, liberates free fatty acids [31]. UCP1 is a membrane protein integrated into the mitochondrial inner membrane. When activated by fatty acids, UCP1 facilitates an increase in the proton conductance of the inner mitochondrial membrane, thereby diminishing proton motive force in the absence of ATP production. Instead of ADP phosphorylation, this process generates heat, which comes at a high energetic cost, thus making BAT an energy-wasting organ [31,32]. During thermogenesis, BAT utilizes several circulating substrates in addition to triglycerides, including glucose, fatty acids, and some amino acids. Accordingly, in addition to increasing energy expenditure, BAT activation could potentially improve certain metabolic parameters (hyperglycemia, dyslipidemia), making BAT activation a potential therapeutic target for obesity and other metabolic diseases [16,28]. BAT activation is most commonly achieved through cold exposure, with pharmacotherapy being a potential alternative, consisting of agents such as sympathomimetics, thyroid hormones, and other compounds (capsaicin and capsinoids, bile acids) [16]. 

**Figure 1:**
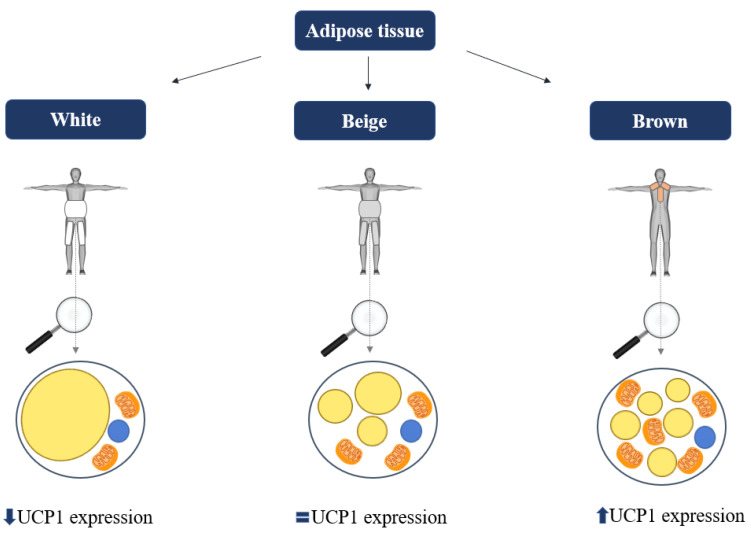
Adipose tissue subtypes—the subtypes differ in the amount of lipid droplets, mitochondrial density, and the level of uncoupling protein 1 (UCP1) expression. Legend: UCP1—uncoupling protein 1.

In addition to WAT and BAT, a third distinct form of adipose tissue can be observed: beige or brite (“brown in white”) adipose tissue [30]. Characteristically, beige adipocytes are an intermediate form between brown and white adipocytes, consisting of multiple lipid droplets that are usually larger than those in brown adipocytes, have more mitochondria than white adipocytes, and also express UCP1 [25]. Beige adipose tissue arises through two different pathways: induction or differentiation of adipocyte progenitor cells or transdifferentiation of mature white adipocytes to brown adipocytes [30]. Figure 1 illustrates the major cellular and anatomical differences between adipose tissue subtypes. Given the relatively small amount of activatable BAT in human adults, the browning of WAT could be a potential strategy to expand the thermogenic potential of adipose tissue mass. Obese individuals, in particular, could greatly benefit from such interventions, considering the fact that WAT accounts for a significant percentage of their total body mass. There are several approaches to WAT browning, including sympathetic activation (β-adrenergic receptor agonism, cold exposure) and capsinoids, all of which have been shown to induce browning in humans in vivo, albeit with varying degrees of effectiveness [16]. 

## 3. Brown Adipose Tissue

### 3.1. The Relationship between BAT and Anthropometric Measures

Initial studies exploring BAT activity demonstrated that BAT is present in both lean and obese individuals; however, BAT activity was observed to be reduced in overweight or obese men [33,34]. This observation was corroborated by the finding that BAT activity was severely blunted in obesity while also associating detectable BAT with metabolic health [35]. Studies also showed that BAT could be recruited after bariatric-surgery-induced weight loss [36,37]. In recent years, on the other hand, studies have taken the initial observations into question; studies have shown that although the prevalence of BAT in obese individuals is indeed lower compared to lean controls, it is still highly prevalent in obesity [17,38]. Furthermore, some research groups observed that BAT metabolic activity and thermogenic capacity seem to remain intact in obesity [17]. In addition, researchers have reported an inverse association between BAT activity and visceral fat mass [17,39]. A common deduction summarizing recent studies of BAT activity in obesity is the association of active BAT with a beneficial metabolic phenotype in obesity [35,38,39,40]. These new findings support research efforts that strive to find strategies that could recruit and activate BAT while also promoting further studies focusing on the metabolic role of BAT in obesity. 

Therefore, the significant correlation between BAT mass, activity, and metabolic health has sparked increased interest in studying its therapeutic potential in the treatment of metabolic diseases and obesity. Its ability to target energy excess per se has provided an attractive avenue to address chronic energy imbalance that can result in adiposity-based chronic diseases. In addition, its theoretical high metabolic activity and the secretion of “batokines” could increase the clearance and utilization of circulating glucose and lipids, thus improving metabolic homeostasis [41,42,43,44]. However, the extent of the maximum possible contribution of human BAT on whole-body thermogenesis, energy expenditure, and nutrient disposal is still not entirely determined. 

### 3.2. Activation of Brown Adipose Tissue with Cold Exposure

To date, cold exposure is the most well-studied physiological stimulus for BAT activation. Therefore, studies deploying acute and chronic cold exposure could offer some perspective for all other interventions aiming for BAT activation. 

During cold exposure, body surface temperature sensors are activated. Neuronal signals are transduced to the thermoregulatory center in the hypothalamus, which leads to instant activation of the sympathetic nervous system. Noradrenaline released from sympathetic nerve endings stimulates brown adipocytes mainly via the β3-adrenergic receptor and triggers cAMP-activated intracellular events. Those include the activation of lipolytic enzymes, therefore, increasing the availability of the substrates for the thermogenesis and activation of UCP1 [42,43,45]. A meta-analysis of ten randomized controlled trials on humans aiming to explore the effects of acute cold exposure on energy expenditure revealed that the energy expenditure was increased following acute cold exposure at between 16 and 19 °C, with a mean difference of 188.43 kal/d (95% CI = 139.73–237.13). In addition, the BAT volume and activity also increased [46]. Furthermore, a recent systematic review identified twenty-two human studies of mostly short durations that tested the use of cold exposure [47]. Most studies found increases in energy expenditure using a variety of measurement techniques [47]. Limitations to the above finding include a study demonstrating an 8.3% decrease in energy expenditure [48] and an additional study finding no effect on energy expenditure after cold stimulation [49]. Furthermore, cold-activated BAT was already reported to increase basal and insulin-stimulated whole-body glucose disposal [50,51,52], and cold-activated glucose uptake in BAT is greater than insulin-stimulated glucose uptake in BAT [53]. In addition, data from observational studies demonstrate the role of human BAT in lipid and fatty acid metabolism [42,54].

## 4. Brown Adipose Tissue as a New Potential Target for Glucagon-like Peptide 1 Receptor Agonists

### 4.1. Glucagon-like Peptide 1 Receptor Agonists in Obesity Management

Glucagon-like peptide 1 (GLP-1) is a peptide hormone primarily secreted by endocrine cells in the distal intestine, alpha cells in the pancreas, and cells in the central nervous system [55]. It is commonly classified as an incretin, a gastrointestinal hormone released after nutrient intake with the capability of glucose-dependently augmenting insulin secretion [56]. GLP-1 takes part in the regulation of glucose homeostasis by interacting with the GLP-1 receptor, which can also be activated by GLP-1RAs [55]. GLP-1RAs enhance glucose-dependent insulin release, reduce gastric emptying, and decrease glucagon secretion, making them effective drugs in type 2 diabetes management [57]. The first GLP-1RA approved by the FDA for type 2 diabetes treatment was exenatide in 2005 [58]. Since then, several other GLP-1RAs have been approved for type 2 diabetes management, available either for subcutaneous injection (exenatide, lixisenatide, liraglutide, dulaglutide, albiglutide, semaglutide), oral administration (semaglutide), or as fixed-dose combinations (liraglutide/insulin degludec, lixisenatide/insulin glargine) [56]. 

Preclinical experiments linked GLP-1 receptor activation to weight loss, and body weight reduction was observed in people with type 2 diabetes treated with liraglutide 1.2 and 1.8 mg once daily [57]. Clinical trials confirmed the efficacy of subcutaneous liraglutide 3.0 mg once daily in reducing body weight in obese and overweight individuals without type 2 diabetes as an adjunct to diet and exercise, demonstrating that liraglutide sustained weight loss and was well tolerated [59,60,61]. Similarly, clinical trials have also shown that subcutaneous semaglutide 2.4 mg once weekly in adults with overweight or obesity and without diabetes, in addition to intensive behavioral therapy and diet, resulted in significant weight loss, which continued when semaglutide treatment was maintained [19,62]. When comparing the effect of once-weekly subcutaneous semaglutide 2.4 mg with once-daily subcutaneous liraglutide 3.0 mg, both as an adjunct to diet and physical activity in obese or overweight persons without diabetes, semaglutide resulted in significantly greater weight loss [18]. 

GLP1-RAs-induced weight loss can be attributed to several proposed mechanisms of action, including reductions in appetite and hunger, alterations in food reward pathways, and improvement in eating control [63]. Semaglutide also markedly reduced 4 h gastric emptying after ingestion of a standardized solid test meal using technetium scintigraphy in women with PCOS and obesity, providing additional insight into the potential peripheral mechanism of reducing energy intake [64].

New exciting treatments for obesity management are emerging, with tirzepatide currently in the spotlight as a promising anti-obesity agent. Tirzepatide is a dual GLP-1 and glucose-dependent insulinotropic peptide (GIP) receptor agonist recently approved by the FDA for treating type 2 diabetes [65]. However, it has been shown that tirzepatide once weekly is effective in providing a substantial and sustained reduction in body weight in obese persons without diabetes [66].

### 4.2. The Impact of GLP-1 Receptor Agonism on Brown Adipose Tissue: Preclinical Studies

Several studies have explored the effect of GLP-1RAs on adipose tissue in animal models, proposing the hypothesis that GLP-1RAs play a significant role in whole-body energy metabolism. Lipid storage in WAT has been shown to directly and potently decrease following GLP-1 infusion into the central nervous systems of mice. These effects were independent of nutrient intake and were partially dependent on functioning sympathetic nervous system signaling. The results implied that the central nervous system GLP-1 signaling directly modulated adipocyte metabolism, thereby decreasing fat storage. Importantly, in diet-induced obese mice, the central nervous system GLP-1 effect on adipocyte metabolism was blunted, suggesting possible obesity-induced resistance of adipocytes to the central nervous system GLP-1 [67]. A further study explored the effect of centrally administered proglucagon-derived peptides (which include GLP-1, glucagon, and oxyntomodulin) in mice, additionally exploring how these compounds affect BAT activity. Intracerebroventricular injection of proglucagon-derived peptides reduced body weight and increased BAT activity. These effects were independent of changes in feeding and correlated with increased activity of sympathetic nerve fibers innervating BAT [68]. 

Moreover, the centrally injected long-acting GLP-1RA (liraglutide) stimulated BAT thermogenesis and adipocyte browning independent of nutrient intake in mice. The mechanism controlling these actions is located at the hypothalamic ventromedial nucleus, with AMPK activation diminishing liraglutide-induced thermogenesis and adipocyte browning [20]. In a further study, the central administration of GLP-1RA exendin-4 in lean mice increased sympathetic outflow towards WAT and BAT, increasing thermogenesis and uptake of triacylglycerol-derived fatty acids in WAT and BAT. In diet-induced obese mice, the effects on WAT were blunted; however, exendin-4 still increased sympathetic outflow towards BAT and increased glucose and triacylglycerol-derived fatty acid uptake by BAT. Most importantly, these effects were accompanied by a reduction in body weight and lower plasma triacylglycerol as well as glucose concentrations [69]. 

The animal studies mentioned above support the notion that central GLP-1 receptor agonism induces weight loss not only by reducing food intake through appetite and hunger reduction but also by increasing energy expenditure, most likely through BAT thermogenesis. However, a study conducted by Kaineder et al. on rats showed that intrahypothalamic administration of liraglutide indeed resulted in significant body weight and fat mass reduction, but the researchers attributed this effect to changes in the hypothalamic melanocortin system rather than increased thermogenesis or adipose tissue browning [70]. 

Recent animal studies have further explored the mechanism by which GLP-1 receptor agonism affects adipose tissue. Intraperitoneal liraglutide was shown to increase BAT oxygen consumption, which was accompanied by the upregulation of UCP-1 protein levels in BAT. Most importantly, liraglutide increased BAT type 2 deiodinase activity, suggesting that it may activate BAT by increasing intracellular thyroid activation and thus facilitating weight loss [71]. Furthermore, a recent study by Gutierrez et al. hypothesized that GLP-1RAs signal through interleukin-6 in adipose tissue. They used a mouse model and administered liraglutide intraperitoneally, demonstrating that liraglutide transiently increased interleukin-6 in mouse circulation while also increasing interleukin-6 receptor signaling in adipose tissue. These effects were accompanied by adipose tissue browning and thermogenesis. Most importantly, interleukin-6-blocking antibodies suppressed the liraglutide-induced increase in thermogenesis, and adipose interleukin-6 receptor knockout mice still displayed liraglutide-induced weight loss but lacked thermogenic adipocyte browning. Thus, the researchers suggested that GLP-1RAs mediate their metabolic effects through transient upregulation of interleukin-6, in turn, activating thermogenesis [72]. These recent animal studies indicate that the mechanism by which GLP-1RAs induce their metabolic effects potentially involves a complex, multi-layered system, and future studies exploring these mechanisms are warranted.

### 4.3. The Impact of GLP-1 Receptor Agonism on Brown Adipose Tissue: Clinical Studies

An initial human study assessing liraglutide’s effect on BAT was conducted by van Eyk et al. and published in 2020 [73]. The primary goal of this study was to investigate the effect of liraglutide treatment on resting energy expenditure (REE) in patients with type 2 diabetes by indirect calorimetry; however, this study also assessed the impact of liraglutide on the BAT fat fraction of the supraclavicular BAT depot using magnetic resonance imaging (MRI). In this double-blind, placebo-controlled clinical trial, 50 participants with type 2 diabetes were randomized to receive either liraglutide (1.8 mg/day) or placebo in addition to standard care for 26 weeks. The mean baseline BMI (SD) was 32.6 (4.4) kg/m^2^ in the liraglutide group and 31.6 (3.4) kg/m^2^ in the placebo group. Treatment with liraglutide for 26 weeks resulted in decreased body weight (−4.3 ± 3.8 kg, *p* < 0.001) compared to placebo (+0.1 ± 2.5 kg, *p* = 0.827). Body weight reduction was already observed after 4 weeks. In addition, treatment with liraglutide was also associated with decreased lean body mass (−2.1 ± 2.9 kg, *p* = 0.003) compared with placebo (−0.2 ± 1.6 kg, *p* = 0.455). Liraglutide treatment was associated with statistically significant reductions in REE after 4 weeks, persisting after 12 weeks, and the reduction tended to be present after 26 weeks (week 26 vs. baseline: −52 ± 128 kcal/day, *p* = 0.071) compared with placebo (week 26 vs. baseline: +44 ± 144 kcal/day, *p* = 0.153). When correcting for lean body mass, the reduction of REE after 4 weeks was still present. In contrast, liraglutide did not significantly decrease REE after 12 and 26 weeks after correcting for lean body mass. Glucose and lipid oxidation rates remained unchanged in both groups. The fat fraction in the supraclavicular BAT depot was measured in 22 participants (10 in the liraglutide group and 12 in the placebo group); treatment with liraglutide for 26 weeks was not associated with fat fraction reduction (−0.4 ± 1.7%, *p* = 0.447) compared to placebo (−0.4 ± 1.4%, *p* = 0.420) [73]. 

Another human study published by Janssen et al. primarily focused on the impact of exenatide on BAT while also exploring the effects of exenatide on body weight, energy expenditure, plasma glucose, and lipid levels [74]. A total of 24 adult participants were included in this single-arm prospective study, divided into a group of 12 Europids and a group of 12 South Asians. Both groups were age and BMI matched and included only non-diabetic male participants, and the baseline mean BMI for all participants was 23.9 ± 0.5 kg/m^2^. The participants received extended release exenatide 2 mg subcutaneously once weekly for 12 weeks. BAT was visualized using a cold-induced ^18^F-FDG-PET/CT scan and a thermoneutral MRI scan before and after treatment with exenatide. Additionally, REE, substrate oxidation and body composition were measured before and after treatment. Exenatide lowered body weight in the total cohort (−1.5 ± 0.4 kg, *p* < 0.01), which was primarily due to lean body mass reduction (−1.1 ± 0.4 kg, *p* < 0.01). However, exenatide use was associated with decreased fat mass only in South Asians (−1.0 ± 0.4 kg, *p* < 0.05), in contrast to Europids (0.2 ± 0.4 kg, *p* < 0.68). When investigating whether an altered energy metabolism determined these weight-lowering effects of exenatide, the results demonstrated that exenatide use did not affect the REE (nor when corrected for lean body mass), substrate oxidation, or respiratory quotient (RQ) in the total cohort, aside from a trend towards lower glucose oxidation rate [74]. When studying the effect of exenatide on BAT using ^18^F-FDG-PET/CT, the researchers observed that exenatide increased the metabolic volume (+28%, *p* < 0.05) and mean standardized uptake value (SUV_mean_) (+11%, *p* < 0.05) of cervical and supraclavicular BAT depots in the total cohort. Similar results were observed when they additionally included the upper mediastinal, axillary, and paravertebral BAT depots. Notably, seasonal variations or changes in body weight or composition could not explain the effects of exenatide on BAT. Furthermore, exenatide did not impact ^18^F-FDG uptake in the subcutaneous or visceral WAT depots. In addition, using MRI to assess supraclavicular adipose tissue before and after treatment with exenatide, the fat fraction (0.745 ± 0.009 vs. 0.745 ± 0.008, *p* = 0.96) and volume (31.0 ± 2.8 mL vs. 30.2 ± 2.9, *p* = 0.22) of this adipose tissue depot remained unchanged in the total cohort. Of note, the researchers observed that the change in fat fraction on MRI scans was negatively correlated with the change in SUV_mean_ on ^18^F-FDG-PET/CT in the supraclavicular adipose tissue [74]. Table 1 provides an overview of the two clinical studies discussed in detail above that assessed GLP-1RAs’ effect on BAT. 

To the best of our knowledge, these are the only already published studies that assessed the effect of GLP1-RAs on BAT using MRI or ^18^F-FDG-PET/CT in humans. In the exenatide study, the researchers observed that exenatide increased the metabolic volume and SUV_mean_ of cervical and supraclavicular BAT depots in the total cohort, with similar results observed in the upper mediastinal, axillary, and paravertebral BAT depots using cold-induced ^18^F-FDG-PET/CT [74]. This significant result supported the thesis that GLP-1RAs contribute to BAT activation in humans. Moreover, exenatide did not impact ^18^F-FDG uptake in the subcutaneous or visceral WAT depots, suggesting that BAT activation in classical BAT depots is a more likely effect of GLP-1RAs than WAT browning [74]. Notably, these results were observed in young, non-diabetic, lean adults with presumably favorable metabolic profiles. However, it is plausible that BAT activity was decreased in individuals with obesity, indicating that BAT plays a role in the setting of obesity [75]. 

Both studies evaluated the supraclavicular BAT depot using MRI [73,74]. Treatment with liraglutide for 26 weeks was not associated with fat fraction reduction compared to placebo. Similarly, treatment with exenatide did not affect the fat fraction and volume in the total cohort. If GLP-1RAs would activate BAT and thus promote intracellular lipid combustion, it would be expected that the fat fraction of BAT would reduce; however, the results of these two studies do not support this hypothesis. A possible explanation for these results could be that the participants were given liraglutide 1.8 mg/day in the liraglutide study, a fair amount lower than the current recommended dose of liraglutide 3.0 mg/day in weight-loss management [76]. It could be that the lower dose was insufficient for substantial BAT activation. Furthermore, it is plausible that BAT was not activated in the supraclavicular adipose tissue depot, which does not exclude BAT activation elsewhere in the body, given the known locations of BAT depots in adults [28]. Lastly, it cannot be excluded that MRI cannot measure a net increase in intracellular lipid combustion after GLP-1RA treatment, given the strict regulation of lipid utilization by activated BAT [74].

Treatment with liraglutide resulted in decreased body weight compared to placebo, which was mainly attributed to decreased lean body mass compared to placebo. Additionally, liraglutide use was associated with reduced REE even when correcting for lean body mass. Similar results were observed in the exenatide study, with exenatide use reducing body weight in the total cohort, primarily due to lean body mass reduction. Furthermore, exenatide use did not affect the REE (nor when corrected for lean body mass) in the total cohort. The results of both studies are in line with the well-documented effects of GLP-1RAs on body weight reduction [77,78]. Interestingly, lean body mass reduction was observed in both studies, with fat-free mass being a major determinant of REE [79]. However, the results showing decreased or unchanged REE in both studies after GLP-1RA treatment contrasted with initial studies, where GLP-1RA treatment tended to increase REE [20,21], especially when adjusted for fat-free mass [20]. This discrepancy could possibly be explained by the fact that the initial studies used different methodologies and treatment periods; however, there is support for the notion that REE decreases after weight loss [80]. Therefore, a plausible explanation for REE reduction after 4 weeks of liraglutide treatment, even when correcting for lean body mass, could be that REE adapts to increase metabolic efficiency in response to decreased food intake, mitigating the loss of body weight [73]. 

When comparing the two studies, it is essential to note the significant differences between them. Firstly, the studies involved different participant groups. Patients with type 2 diabetes of both sexes and with a mean BMI in the range for obesity classification were recruited in the liraglutide study [73]. In contrast, the participants in the exenatide study [74] were only non-diabetic males with an average BMI within the healthy weight range. Furthermore, the participants in the liraglutide study were older than those in the exenatide study. Secondly, there were inherent differences in study design, with the liraglutide study being a double-blind, placebo-controlled clinical trial, while, on the other hand, the exenatide study was a single-arm prospective study without a placebo group. Lastly, study duration needs to be considered, with the liraglutide study taking place over 26 weeks, whereas the exenatide study was only 12 weeks long.

**Figure 2:**
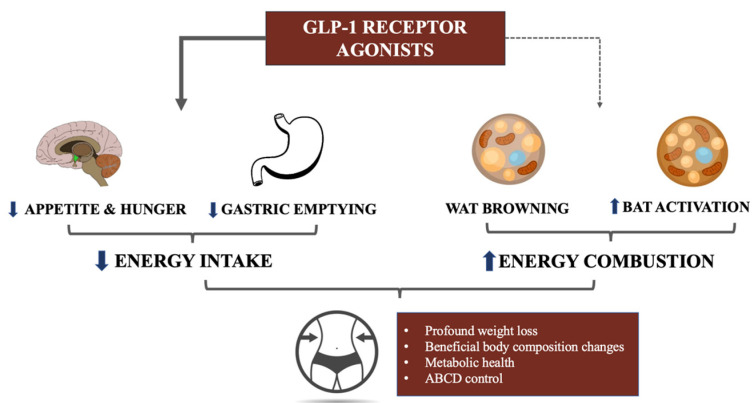
The potential mechanisms of GLP-1RAs action: from well-established effects in reducing energy intake combined to their potential role in increasing energy expenditure. Legend: WAT—white adipose tissue; BAT—brown adipose tissue; ABCD—adipose-based chronic disease.

Figure 2 summarizes the potential multilayered mechanism of GLP-1RAs action.

## 5. Quantifying Brown Adipose Tissue and Its Function in Humans

The development of reliable and accessible BAT visualization and activity measurement methods is crucial to better explore the impact of GLP-1 receptor agonism on BAT and assess the potential long-term effects of BAT activation on cardiometabolic health improvement induced by GLP-1RAs. 

^18^F-FDG-PET/CT is the most commonly used and best-established method for activated BAT visualization in humans [81]; however, it requires BAT to be activated by stimulators, such as cold exposure or drug treatment [82]. Obese individuals with or without type 2 diabetes, who are candidates for GLP-1RA treatment and greatly benefit from such therapy, are prone to insulin resistance, which significantly hampers ^18^F-FDG uptake and thus makes imaging with ^18^F-FDG-PET/CT inconclusive [83,84]. An alternative radiolabeled tracer, [123I]metaiodobenzylguanidine ([123I]MIBG), is a norepinephrine analog routinely used for imaging of tumors of the sympathetic nervous system and reflects sympathetic stimulation and activation of BAT [85,86]. While not affected by insulin resistance, the uptake in the target tissues may be altered by a range of medications. In addition, as a single photon emitting tracer, it offers lower spatial resolution in comparison to positron emitting alternatives. Structurally similar, positron-emitting derivatives, as well as alternatives sharing similar uptake pathways ([124I]MIBG, [18F]MFBG, [18F]FDOPA [87]), are already entering the clinical arena and are expected to overcome this disadvantage. It is also worth noting that molecular imaging methods using radionuclide-labeled tracers expose the participants to ionizing radiation, making the justification of such studies in healthy individuals difficult, especially when considering longitudinal studies requiring multiple scans. However, recent developments in PET/CT and PET/MR technology will likely lead to significant (up to 10-fold) reductions in radiation exposure while, at the same time, offering dynamic total-body metabolic assessments in real-time, as well as unprecedented possibilities of longitudinal follow-up [88,89]. 

BAT can be distinguished from WAT due to its higher water content and corresponding lower fat fraction. MRI utilizes the fact that the fat fraction of BAT decreases due to the combustion of intracellular triglyceride stores upon BAT activation [90,91]. Using MRI and in-house developed water–fat separation algorithms, the researchers in the liraglutide and exenatide studies constructed fat fraction maps of the supraclavicular adipose tissue depot [73,74]. Therefore, MRI offers an alternative way for BAT imaging without exposing the participants to unnecessary ionizing radiation. However, the identification of BAT based on MRI fat fraction measurements is challenging and prone to variation with age, BMI, diet, and external temperature. Furthermore, MRI scanning is time-consuming and often requires sedation [75]. 

In recent years, infrared thermography (IRT) has gained traction as a promising imaging modality for BAT imaging. IRT is non-invasive, cost-efficient, and quick, possibly allowing for large-scale studies of BAT in a plethora of patient populations, including children [92]. Recent studies support IRT as a promising method for studying BAT activity [93], and ongoing research is making progress in making IRT a reliable BAT research tool [94]. However, IRT still requires protocol standardization to be used on a broader scale.

## 6. Conclusions

In conclusion, BAT presence is associated with beneficial metabolic phenotype in humans. BAT can be pharmacologically activated. However, there is limited evidence that GLP-1RAs contribute to BAT activation in preclinical models and even more scarce data in humans. Further research is warranted to explore the impact of GLP1-RAs on BAT activation in individuals with obesity, possibly with novel non-invasive imaging modalities on a larger scale. 

## Figures and Tables

**Table 1:** Overview of the clinical studies assessing GLP-1RAs effect on BAT.

Study	GLP-1RA (Dose)	Duration (Weeks)	Participant Characteristics (Number of Participants)	Body Weight Difference (kg)	Lean Body Mass Difference (kg)	BAT Difference Using MRI	BAT Difference Using ^18^F-FDG-PET/CT
Van Eyk et al. [73]	liraglutide (1.8 mg/day)	26	Type 2 diabetes (50)	−4.3 ± 3.8	−2.1 ± 2.9	n.s.	/
Janssen et al. [74]	exenatide (2 mg/week)	12	Non-diabetic males (24)	−1.5 ± 0.4	−1.1 ± 0.4	n.s.	+28% MV +11% SUV_mean_

Legend: GLP-1RA—Glucagon-like peptide-1 receptor agonist; BAT—brown adipose tissue; MRI—magnetic resonance imaging; ^18^F-FDG-PET/CT—fluorine-18 fluorodeoxyglucose positron emission tomography coupled with CT; n.s.—not significant; MV—metabolic volume; SUV_mean_—mean standardized uptake value.

## Data Availability

Not applicable.

## References

[1] Obesity and Overweight. https://www.who.int/news-room/fact-sheets/detail/obesity-and-overweight.

[2] World Health Organization, Regional Office for Europe (2022). WHO European Regional Obesity Report. https://apps.who.int/iris/handle/10665/353747.

[3] Flegal K.M., Kit B.K., Orpana H., Graubard B.I. (2013). Association of all-cause mortality with overweight and obesity using standard body mass index categories: A systematic review and meta-analysis. JAMA.

[4] Di Angelantonio E., Bhupathiraju S.N., Wormser D., Gao P., Kaptoge S., de Gonzalez A.B., Cairns B.J., Huxley R., Jackson C.L., Joshy G. (2016). Body-mass index and all-cause mortality: Individual-participant-data meta-analysis of 239 prospective studies in four continents. Lancet.

[5] Haslam D.W., James W.P.T. (2005). Obesity. Lancet.

[6] Blüher M. (2019). Obesity: Global epidemiology and pathogenesis. Nat. Rev. Endocrinol..

[7] Calle E.E., Rodriguez C., Walker-Thurmond K., Thun M.J. (2003). Overweight, Obesity, and Mortality from Cancer in a Prospectively Studied Cohort of U.S. Adults. N. Engl. J. Med..

[8] Kim S.-R., Kim H.-N., Song S.-W. (2020). Associations Between Mental Health, Quality of Life, and Obesity/Metabolic Risk Phenotypes. Metab. Syndr. Relat. Disord..

[9] Le Strat Y., Melchior M., Gorwood P., Tebeka S., Dubertret C. (2020). The role of comorbidity in the association of obesity with unemployment and disability. Ann. Epidemiol..

[10] Garvey W.T. (2021). New Horizons. A New Paradigm for Treating to Target with Second-Generation Obesity Medications. J. Clin. Endocrinol. Metab..

[11] Garvey W.T., Mechanick J.I., Brett E.M., Garber A.J., Hurley D.L., Jastreboff A.M., Nadolsky K., Pessah-Pollack R., Plodkowski R., Reviewers of the AACE/ACE Obesity Clinical Practice Guidelines (2016). American association of clinical endocrinologists and american college of endocrinology comprehensive clinical practice guidelines for medical care of patients with obesity. Endocr. Pract..

[12] Willoughby D., Hewlings S., Kalman D. (2018). Body Composition Changes in Weight Loss: Strategies and Supplementation for Maintaining Lean Body Mass, a Brief Review. Nutrients.

[13] Kawai T., Autieri M.V., Scalia R. (2021). Adipose tissue inflammation and metabolic dysfunction in obesity. Am. J. Physiol.-Cell Physiol..

[14] Longo M., Zatterale F., Naderi J., Parrillo L., Formisano P., Raciti G.A., Beguinot F., Miele C. (2019). Adipose tissue dysfunction as determinant of obesity-associated metabolic complications. Int. J. Mol. Sci..

[15] Marlatt K.L., Ravussin E. (2017). Brown Adipose Tissue: An Update on Recent Findings. Curr. Obes. Rep..

[16] McNeill B.T., Suchacki K.J., Stimson R.H. (2021). MECHANISMS IN ENDOCRINOLOGY: Human brown adipose tissue as a therapeutic target: Warming up or cooling down?. Eur. J. Endocrinol..

[17] Kulterer O.C., Herz C.T., Prager M., Schmöltzer C., Langer F.B., Prager G., Marculescu R., Kautzky-Willer A., Hacker M., Haug A.R. (2022). Brown Adipose Tissue Prevalence Is Lower in Obesity but Its Metabolic Activity Is Intact. Front. Endocrinol..

[18] Rubino D.M., Greenway F.L., Khalid U., O’neil P.M., Rosenstock J., Sørrig R., Wadden T.A., Wizert A., Garvey W.T., STEP 8 Investigators (2022). Effect of Weekly Subcutaneous Semaglutide vs Daily Liraglutide on Body Weight in Adults with Overweight or Obesity without Diabetes: The STEP 8 Randomized Clinical Trial. JAMA.

[19] Wadden T.A., Bailey T.S., Billings L.K., Davies M., Frias J.P., Koroleva A., Lingvay I., O’neil P.M., Rubino D.M., Skovgaard D. (2021). Effect of Subcutaneous Semaglutide vs Placebo as an Adjunct to Intensive Behavioral Therapy on Body Weight in Adults with Overweight or Obesity: The STEP 3 Randomized Clinical Trial. JAMA.

[20] Beiroa D., Imbernon M., Gallego R., Senra A., Herranz D., Villarroya F., Serrano M., Fernø J., Salvador J., Escalada J. (2014). GLP-1 agonism stimulates brown adipose tissue thermogenesis and browning through hypothalamic AMPK. Diabetes.

[21] Horowitz M., Flint A., Jones K.L., Hindsberger C., Rasmussen M.F., Kapitza C., Doran S., Jax T., Zdravkovic M., Chapman I.M. (2012). Effect of the once-daily human GLP-1 analogue liraglutide on appetite, energy intake, energy expenditure and gastric emptying in type 2 diabetes. Diabetes Res. Clin. Pract..

[22] Coelho M., Oliveira T., Fernandes R. (2013). Biochemistry of adipose tissue: An endocrine organ. Arch. Med. Sci..

[23] Chait A., den Hartigh L.J. (2020). Adipose Tissue Distribution, Inflammation and Its Metabolic Consequences, Including Diabetes and Cardiovascular Disease. Front. Cardiovasc. Med..

[24] Heinonen S., Jokinen R., Rissanen A., Pietiläinen K.H. (2019). White adipose tissue mitochondrial metabolism in health and in obesity. Obes. Rev..

[25] Jung S.M., Sanchez-Gurmaches J., Guertin D.A. (2019). Brown adipose tissue development and metabolism. Handbook of Experimental Pharmacology.

[26] Kwok K.H.M., Lam K.S.L., Xu A. (2016). Heterogeneity of white adipose tissue: Molecular basis and clinical implications. Exp. Mol. Med..

[27] Hany T.F., Gharehpapagh E., Kamel E.M., Buck A., Himms-Hagen J., von Schulthess G.K. (2002). Brown adipose tissue: A factor to consider in symmetrical tracer uptake in the neck and upper chest region. Eur. J. Nucl. Med..

[28] Leitner B., Huang S., Brychta R.J., Duckworth C.J., Baskin A.S., McGehee S., Tal I., Dieckmann W., Gupta G., Kolodny G.M. (2017). Mapping of human brown adipose tissue in lean and obese young men. Proc. Natl. Acad. Sci. USA.

[29] Kahn C.R., Wang G., Lee K.Y. (2019). Altered adipose tissue and adipocyte function in the pathogenesis of metabolic syndrome. J. Clin. Investig..

[30] Kuryłowicz A., Puzianowska-Kuźnicka M. (2020). Induction of Adipose Tissue Browning as a Strategy to Combat Obesity. Int. J. Mol. Sci..

[31] Li Y., Fromme T. (2022). Uncoupling Protein 1 Does Not Produce Heat without Activation. Int. J. Mol. Sci..

[32] Crichton P.G., Lee Y., Kunji E.R. (2017). The molecular features of uncoupling protein 1 support a conventional mitochondrial carrier-like mechanism. Biochimie.

[33] Van Marken Lichtenbelt W.D., Vanhommerig J.W., Smulders N.M., Drossaerts J.M.A.F.L., Kemerink G.J., Bouvy N.D., Schrauwen P., Teule G.J.J. (2009). Cold-Activated Brown Adipose Tissue in Healthy Men. N. Engl. J. Med..

[34] Virtanen K.A., Lidell M.E., Orava J., Heglind M., Westergren R., Niemi T., Taittonen M., Laine J., Savisto N.-J., Enerbäck S. (2009). Functional Brown Adipose Tissue in Healthy Adults. N. Engl. J. Med..

[35] Orava J., Nuutila P., Noponen T., Parkkola R., Viljanen T., Enerbäck S., Rissanen A., Pietiläinen K., Virtanen K.A. (2013). Blunted metabolic responses to cold and insulin stimulation in brown adipose tissue of obese humans. Obesity.

[36] Vijgen G.H.E.J., Bouvy N.D., Teule G.J.J., Brans B., Hoeks J., Schrauwen P., van Marken Lichtenbelt W.D. (2012). Increase in brown adipose tissue activity after weight loss in morbidly obese subjects. J. Clin. Endocrinol. Metab..

[37] Rachid B., Van De Sande-Lee S., Rodovalho S., Folli F., Beltramini G.C., Morari J., Amorim B.J., Pedro T., Ramalho A.F., Bombassaro B. (2015). Distinct regulation of hypothalamic and brown/beige adipose tissue activities in human obesity. Int. J. Obes..

[38] Mihalopoulos N.L., Yap J.T., Beardmore B., Holubkov R., Nanjee M.N., Hoffman J.M. (2020). Cold-Activated Brown Adipose Tissue is Associated with Less Cardiometabolic Dysfunction in Young Adults with Obesity. Obesity.

[39] Herz C.T., Kulterer O.C., Prager M., Schmöltzer C., Langer F.B., Prager G., Marculescu R., Kautzky-Willer A., Hacker M., Haug A.R. (2021). Active Brown Adipose Tissue Is Associated with a Healthier Metabolic Phenotype in Obesity. Diabetes.

[40] Wibmer A.G., Becher T., Eljalby M., Crane A., Andrieu P.C., Jiang C.S., Vaughan R., Schöder H., Cohen P. (2021). Brown adipose tissue is associated with healthier body fat distribution and metabolic benefits independent of regional adiposity. Cell Rep. Med..

[41] Verduci E., Calcaterra V., Di Profio E., Fiore G., Rey F., Magenes V.C., Todisco C.F., Carelli S., Zuccotti G.V. (2021). Brown adipose tissue: New challenges for prevention of childhood obesity. A narrative review. Nutrients.

[42] da Silva C.P.V., Hernández-Saavedra D., White J.D., Stanford K.I. (2019). Cold and exercise: Therapeutic tools to activate brown adipose tissue and combat obesity. Biology.

[43] Saito M., Yoneshiro T., Matsushita M. (2016). Activation and recruitment of brown adipose tissue by cold exposure and food ingredients in humans. Best Pract. Res. Clin. Endocrinol. Metab..

[44] Fernández-Verdejo R., Marlatt K.L., Ravussin E., Galgani J.E. (2019). Contribution of brown adipose tissue to human energy metabolism. Mol. Asp. Med..

[45] Scheel A.K., Espelage L., Chadt A. (2022). Many Ways to Rome: Exercise, Cold Exposure and Diet—Do They All Affect BAT Activation and WAT Browning in the Same Manner?. Int. J. Mol. Sci..

[46] Huo C., Song Z., Yin J., Zhu Y., Miao X., Qian H., Wang J., Ye L., Zhou L. (2022). Effect of Acute Cold Exposure on Energy Metabolism and Activity of Brown Adipose Tissue in Humans: A Systematic Review and Meta-Analysis. Front. Physiol..

[47] Perez L.C., Perez L.T., Nene Y., Umpierrez G.E., Davis G.M., Pasquel F.J. (2022). Interventions associated with brown adipose tissue activation and the impact on energy expenditure and weight loss: A systematic review. Front. Endocrinol..

[48] Schlögl M., Piaggi P., Thiyyagura P., Reiman E.M., Chen K., Lutrin C., Krakoff J., Thearle M.S. (2013). Overfeeding over 24 hours does not activate brown adipose tissue in humans. J. Clin. Endocrinol. Metab..

[49] Loh R.K.C., Formosa M.F., Eikelis N., Bertovic D.A., Anderson M.J., Barwood S.A., Nanayakkara S., Cohen N.D., La Gerche A., Reutens A.T. (2018). Pioglitazone reduces cold-induced brown fat glucose uptake despite induction of browning in cultured human adipocytes: A randomised, controlled trial in humans. Diabetologia.

[50] Hanssen M.J.W., Hoeks J., Brans B., Van Der Lans A.A.J.J., Schaart G., Van Den Driessche J.J., Jörgensen J.A., Boekschoten M.V., Hesselink M.K.C., Havekes B. (2015). Short-term cold acclimation improves insulin sensitivity in patients with type 2 diabetes mellitus. Nat. Med..

[51] Chondronikola M., Volpi E., Børsheim E., Porter C., Annamalai P., Enerbäck S., Lidell M.E., Saraf M.K., Labbe S.M., Hurren N.M. (2014). Brown adipose tissue improves whole-body glucose homeostasis and insulin sensitivity in humans. Diabetes.

[52] Matsushita M., Yoneshiro T., Aita S., Kameya T., Sugie H., Saito M. (2013). Impact of brown adipose tissue on body fatness and glucose metabolism in healthy humans. Int. J. Obes..

[53] Orava J., Nuutila P., Lidell M.E., Oikonen V., Noponen T., Viljanen T., Scheinin M., Taittonen M., Niemi T., Enerbäck S. (2011). Different Metabolic Responses of Human Brown Adipose Tissue to Activation by Cold and Insulin. Cell Metab..

[54] Ruiz J.R., Martinez Tellez B., Sanchez-Delgado G., Osuna-Prieto F.J., Rensen P.C.N., Boon M.R. (2018). Role of Human Brown Fat in Obesity, Metabolism and Cardiovascular Disease: Strategies to Turn Up the Heat. Prog. Cardiovasc. Dis..

[55] Zhao X., Wang M., Wen Z., Lu Z., Cui L., Fu C., Xue H., Liu Y., Zhang Y. (2021). GLP-1 Receptor Agonists: Beyond Their Pancreatic Effects. Front. Endocrinol..

[56] Nauck M.A., Quast D.R., Wefers J., Meier J.J. (2020). GLP-1 receptor agonists in the treatment of type 2 diabetes—State-of-the-art. Mol. Metab..

[57] Drucker D.J. (2021). GLP-1 physiology informs the pharmacotherapy of obesity. Mol. Metab..

[58] Sheahan K.H., Wahlberg E.A., Gilbert M.P. (2019). An overview of GLP-1 agonists and recent cardiovascular outcomes trials. Postgrad. Med. J..

[59] Astrup A., Carraro R., Finer N., Harper A., Kunesova M., Lean M.E.J., Niskanen L., Rasmussen M.F., Rissanen A., Rössner S. (2011). Safety, tolerability and sustained weight loss over 2 years with the once-daily human GLP-1 analog, liraglutide. Int. J. Obes..

[60] Pi-Sunyer X., Astrup A., Fujioka K., Greenway F., Halpern A., Krempf M., Lau D.C.W., Le Roux C.W., Ortiz R.V., Jensen C.B. (2015). A Randomized, Controlled Trial of 3.0 mg of Liraglutide in Weight Management. N. Engl. J. Med..

[61] Wadden T.A., Hollander P., Klein S., Niswender K., Woo V., Hale P.M., Aronne L. (2013). Weight maintenance and additional weight loss with liraglutide after low-calorie-diet-induced weight loss: The SCALE Maintenance randomized study. Int. J. Obes..

[62] Rubino D., Abrahamsson N., Davies M., Hesse D., Greenway F.L., Jensen C., Lingvay I., Mosenzon O., Rosenstock J., Rubio M.A. (2021). Effect of Continued Weekly Subcutaneous Semaglutide vs Placebo on Weight Loss Maintenance in Adults with Overweight or Obesity. The STEP 4 Randomized Clinical Trial. JAMA.

[63] Ard J., Fitch A., Fruh S., Herman L. (2021). Weight Loss and Maintenance Related to the Mechanism of Action of Glucagon-Like Peptide 1 Receptor Agonists. Adv. Ther..

[64] Jensterle M., Ferjan S., Ležaič L., Sočan A., Goričar K., Zaletel K., Janez A. (2023). Semaglutide delays 4-hour gastric emptying in women with polycystic ovary syndrome and obesity. Diabetes Obes. Metab..

[65] Chavda V.P., Ajabiya J., Teli D., Bojarska J., Apostolopoulos V. (2022). Tirzepatide, a New Era of Dual-Targeted Treatment for Diabetes and Obesity: A Mini-Review. Molecules.

[66] Jastreboff A.M., Aronne L.J., Ahmad N.N., Wharton S., Connery L., Alves B., Kiyosue A., Zhang S., Liu B., Bunck M.C. (2022). Tirzepatide Once Weekly for the Treatment of Obesity. N. Engl. J. Med..

[67] Nogueiras R., Pérez-Tilve D., Veyrat-Durebex C., Morgan D.A., Varela L., Haynes W.G., Patterson J.T., Disse E., Pfluger P.T., López M. (2009). Direct control of peripheral lipid deposition by cns glp-1 receptor signaling is mediated by the sympathetic nervous system and blunted in diet-induced obesity. J. Neurosci..

[68] Lockie S.H., Heppner K.M., Chaudhary N., Chabenne J.R., Morgan D.A., Veyrat-Durebex C., Ananthakrishnan G., Rohner-Jeanrenaud F., Drucker D.J., DiMarchi R. (2012). Direct control of brown adipose tissue thermogenesis by central nervous system glucagon-like peptide-1 receptor signaling. Diabetes.

[69] Kooijman S., Wang Y., Parlevliet E.T., Boon M.R., Edelschaap D., Snaterse G., Pijl H., Romijn J.A., Rensen P.C.N. (2015). Central GLP-1 receptor signalling accelerates plasma clearance of triacylglycerol and glucose by activating brown adipose tissue in mice. Diabetologia.

[70] Kaineder K., Birngruber T., Rauter G., Obermüller B., Eichler J., Münzker J., Al-Zoughbi W., Mautner S., Torekov S., Hartmann B. (2017). Chronic intrahypothalamic rather than subcutaneous liraglutide treatment reduces body weight gain and stimulates the melanocortin receptor system. Int. J. Obes..

[71] Oliveira F.C.B., Bauer E.J., Ribeiro C.M., Pereira S.A., Beserra B.T.S., Wajner S.M., Maia A.L., Neves F.A.R., Coelho M.S., Amato A.A. (2022). Liraglutide Activates Type 2 Deiodinase and Enhances β3-Adrenergic-Induced Thermogenesis in Mouse Adipose Tissue. Front. Endocrinol..

[72] Gutierrez A.D., Gao Z., Hamidi V., Zhu L., Andre K.B.S., Riggs K., Ruscheinsky M., Wang H., Yu Y., Miller C. (2022). Anti-diabetic effects of GLP1 analogs are mediated by thermogenic interleukin-6 signaling in adipocytes. Cell Rep. Med..

[73] van Eyk H.J., Paiman E., Bizino M.B., Ijzermans S.L., Kleiburg F., Boers T.G., Rappel E.J., Burakiewicz J., Kan H.E., Smit J.W. (2019). Liraglutide decreases energy expenditure and does not affect the fat fraction of supraclavicular brown adipose tissue in patients with type 2 diabetes. Nutr. Metab. Cardiovasc. Dis..

[74] Janssen L.G., Nahon K.J., Bracké K.F., Broek D.V.D., Smit R., Mishre A.S.S., Koorneef L.L., Martinez-Tellez B., Burakiewicz J., Kan H.E. (2020). Twelve weeks of exenatide treatment increases [18F]fluorodeoxyglucose uptake by brown adipose tissue without affecting oxidative resting energy expenditure in nondiabetic males. Metabolism.

[75] Singh R., Barrios A., Dirakvand G., Pervin S. (2021). Human brown adipose tissue and metabolic health: Potential for therapeutic avenues. Cells.

[76] Perdomo C.M., Cohen R.V., Sumithran P., Clément K., Frühbeck G. (2023). Contemporary medical, device, and surgical therapies for obesity in adults. Lancet.

[77] Iqbal J., Wu H., Hu N., Zhou Y., Li L., Xiao F., Wang T., Jiang H., Xu S., Huang B. (2022). Effect of glucagon-like peptide-1 receptor agonists on body weight in adults with obesity without diabetes mellitus—A systematic review and meta-analysis of randomized control trials. Obes. Rev..

[78] Vilsbøll T., Christensen M., Junker A.E., Knop F.K., Gluud L.L. (2012). Effects of glucagon-like peptide-1 receptor agonists on weight loss: Systematic review and meta-analyses of randomised controlled trials. BMJ.

[79] Cunningham J. (1991). Original Body composition a synthetic review general prediction of energy expenditure: As a determinant and a proposed. Am. J. Clin. Nutr..

[80] Rosenbaum M., Hirsch J., Gallagher D.A., Leibel R.L. (2008). Long-term persistence of adaptive thermogenesis in subjects who have maintained a reduced body weight. Am. J. Clin. Nutr..

[81] Chen K.Y., Cypess A.M., Laughlin M.R., Haft C.R., Hu H.H., Bredella M.A., Enerbäck S., Kinahan P.E., Lichtenbelt W.V.M., Lin F.I. (2016). Brown Adipose Reporting Criteria in Imaging STudies (BARCIST 1.0): Recommendations for Standardized FDG-PET/CT Experiments in Humans. Cell Metab..

[82] Yang J., Zhang H., Parhat K., Xu H., Li M., Wang X., Ran C. (2021). Molecular Imaging of Brown Adipose Tissue Mass. Int. J. Mol. Sci..

[83] Wu H., Ballantyne C.M. (2020). Metabolic Inflammation and Insulin Resistance in Obesity. Circ. Res..

[84] Schilperoort M., Hoeke G., Kooijman S., Rensen P.C. (2016). Relevance of lipid metabolism for brown fat visualization and quantification. Curr. Opin. Infect. Dis..

[85] Admiraal W.M., Holleman F., Bahler L., Soeters M.R., Hoekstra J.B., Verberne H.J. (2013). Combining ^123^I-Metaiodobenzylguanidine SPECT/CT and ^18^F-FDG PET/CT for the Assessment of Brown Adipose Tissue Activity in Humans during Cold Exposure. J. Nucl. Med..

[86] Crandall J.P., Wahl R.L. (2021). Perspectives on Brown Adipose Tissue Imaging: Insights from Preclinical and Clinical Observations from the Last and Current Century. J. Nucl. Med..

[87] Samim A., Tytgat G.A., Bleeker G., Wenker S.T., Chatalic K.L., Poot A.J., Tolboom N., van Noesel M.M., Lam M.G., de Keizer B. (2021). Nuclear medicine imaging in neuroblastoma: Current status and new developments. J. Pers. Med..

[88] Chondronikola M., Sarkar S. (2020). Total-body PET Imaging: A New Frontier for the Assessment of Metabolic Disease and Obesity. PET Clin..

[89] Katal S., Eibschutz L.S., Saboury B., Gholamrezanezhad A., Alavi A. (2022). Advantages and Applications of Total-Body PET Scanning. Diagnostics.

[90] Stahl V., Maier F., Freitag M.T., Floca R.O., Berger M.C., Umathum R., Diaz M.B., Herzig S., Weber M.-A., Dimitrakopoulou-Strauss A. (2016). *In vivo* assessment of cold stimulation effects on the fat fraction of brown adipose tissue using DIXON MRI. J. Magn. Reson. Imaging.

[91] Khedoe P.P.S.J., Hoeke G., Kooijman S., Dijk W., Buijs J.T., Kersten S., Havekes L.M., Hiemstra P.S., Berbée J.F.P., Boon M.R. (2015). Brown adipose tissue takes up plasma triglycerides mostly after lipolysis. J. Lipid Res..

[92] Law J., Chalmers J., Morris D.E., Robinson L., Budge H., Symonds M.E. (2018). The use of infrared thermography in the measurement and characterization of brown adipose tissue activation. Temperature.

[93] Sun L., Verma S., Michael N., Chan S.P., Yan J., Sadananthan S.A., Camps S.G., Goh H.J., Govindharajulu P., Totman J. (2019). Brown Adipose Tissue: Multimodality Evaluation by PET, MRI, Infrared Thermography, and Whole-Body Calorimetry (TACTICAL-II). Obesity.

[94] Leow M.K.S. (2023). Brown fat detection by infrared thermography—An invaluable research methodology with noteworthy uncertainties confirmed by a mathematical proof. Endocrinol. Diabetes Metab..

